# Inverse Correlation Between Left Atrial Appendage Function and CHA_2_DS_2_-VASc Score in Patients with Atrial Flutter

**DOI:** 10.1038/s41598-019-54505-3

**Published:** 2019-11-28

**Authors:** Mei-Yao Wu, Yen-Nien Lin, Hung-Pin Wu, Ying-Ying Huang, Jan-Yow Chen, Kuo-Hung Lin, Kuan-Cheng Chang

**Affiliations:** 1 School of Post Baccalaureate Chinese Medicine, China Medical University, Taichung, Taiwan; 2 Department of Chinese Medicine, China Medical University Hospital, Taichung, Taiwan; 3 Division of Cardiovascular Medicine, Department of Medicine, China Medical University Hospital, Taichung, Taiwan; 4 Cardiovascular Research Laboratory, China Medical University Hospital, Taichung, Taiwan; 5 Graduate Institute of Biomedical Sciences, China Medical University, Taichung, Taiwan

**Keywords:** Atrial fibrillation, Risk factors

## Abstract

Impaired left atrial appendage ejection fraction (LAA-EF) and peak LAA flow velocity (LAA-FV) are associated with high thromboembolic risks in patients with atrial fibrillation (AF). Herein, we examined LAA function among patients with atrial flutter (AFL) stratified by the CHA_2_DS_2_-VASc score using transesophageal echocardiography (TEE). Of 231 consecutive patients with typical AFL, 84 who fulfilled the inclusion criteria were enrolled. Among them, 57 had ongoing AFL and were divided into the isolated AFL (n = 38) and AFL with paroxysmal AF (PAF) (n = 19) groups, depending on whether they had sporadic AF before TEE. The remaining 27 patients with spontaneous sinus rhythm during TEE were designated as controls. Both the LAA-FV (31.9 cm/s vs. 51.5 cm/s, P = 0.004) and LAA-EF (28.4% vs. 36.5%, P = 0.024) measured during AFL were significantly lower in the AFL + PAF group than in the isolated AFL group. Significant inverse correlations between the CHA_2_DS_2_-VASc score and LAA-EF were identified in the AFL (P = 0.008) and AFL + PAF (P = 0.032) groups. We observed progressive LAA dysfunction in patients with AFL + PAF compared with that in patients with isolated AFL, and the LAA-EF was inversely correlated with the CHA_2_DS_2_-VASc score in these patients. Our findings may have implications on the application of thromboprophylactic therapy in patients with AFL.

## Introduction

Atrial flutter (AFL) is characterized as an organized atrial rhythm with an atrial rate between 250 and 350 beats/minute. It can present alone or coexist with atrial fibrillation (AF). AF is associated with a five-fold increase in the risk of cardiogenic thromboembolic events, and this risk can be clinically evaluated using the CHA_2_DS_2_-VASc score^1^.

Patients with AFL are considered to have equivalent thromboembolic risks similar to patients with AF based on limited data^2,3^. However, these patients, in general, may not be in the same prothrombotic state as those with AF^2^. A recent large-scale retrospective study demonstrated that patients with AFL have a lower incidence of left atrial (LA) appendage (LAA) thrombi and higher LAA emptying velocity than those with AF^4^. Lin *et al*.^4^ further showed that patients with AFL had a lower ischemic stroke risk and mortality rate than those with AF. Therefore, more data are needed to delineate thromboembolic risk in patients with AFL.

The LAA is the major location of intracardiac thrombus formation in patients with nonvalvular AF^5^. Several parameters evaluated on transesophageal echocardiography (TEE), including LA dilatation, LAA thrombi, LA spontaneous echo contrast (SEC), impaired LAA ejection fraction (LAA-EF), and low LAA flow velocities, have been shown to be highly correlated with an increase in thromboembolic events in patients with AF^6,7^. Unlike patients with AF, there are relatively fewer data on LAA function and thrombi in relation to the thromboembolic risk in patients with AFL, and some of these data appear contradictory. Sakurai *et al*.^2^ reported that patients with AFL had well-preserved LAA peak flow velocities compared with those with AF, suggesting a lower thromboembolic risk in AFL. However, Cresti *et al*.^6^ showed that the incidence of LAA thrombi and LA SEC in patients with AFL was similar to that in patients with AF, indicating an equivalent thromboembolic risk between patients with AFL and those with AF. To further stratify the risk of systemic thromboembolism in AFL, we examined LAA function among patients with isolated AFL and AFL with sporadic AF using TEE during AFL.

## Methods

**Figure 1:**
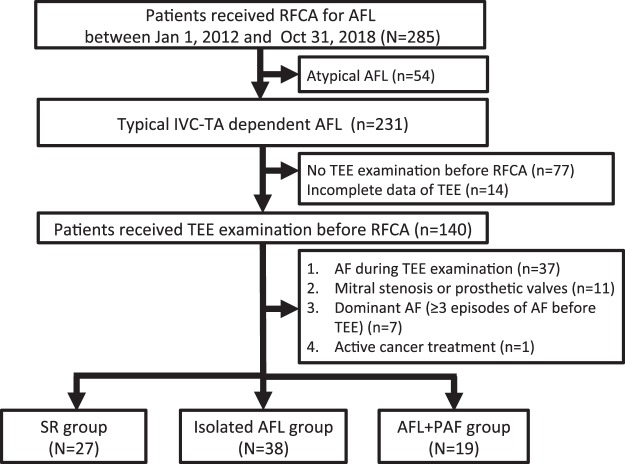
Study population flowchart diagram. Between January 1, 2012 and October 31, 2018, 231 patients who received radiofrequency catheter ablation for typical inferior vena cava-tricuspid isthmus-dependent AFL were enrolled. After excluding patients who met the exclusion criteria, a total of 84 patients constituted the study population for analysis. Of these 84 patients, there were 27 in the SR group, 38 in the isolated AFL group, and 19 in the AFL + PAF group. AFL, atrial flutter; SR, sinus rhythm; PAF, paroxysmal atrial fibrillation.

We conducted a retrospective study of patients with typical inferior vena cava-tricuspid annulus (IVC-TA) isthmus-dependent AFL who underwent radiofrequency catheter ablation (RFCA) between January 1, 2012 and October 31, 2018 to assess the LAA function by using TEE. IVC-TA-dependent AFL was confirmed via an electrophysiological study during RFCA. TEE was typically performed on the same day or 1 day prior to RFCA. Patients who did not receive TEE examination before RFCA were excluded (Fig. 1). We also excluded patients with mitral stenosis, prosthetic mitral or aortic valves, incomplete data on TEE, malignancies receiving active cancer treatment, and dominant AF (≥3 episodes of ECG-documented AF before TEE). For patients who underwent ≥2 RFCAs during the follow-up period, we analyzed their data from only the first RFCA procedure.

For exclusion, patients needed to have an ongoing AFL rhythm for ≥1 day (median: 1 day, interquartile range [IQR]: 1–6.5 days) before TEE examination. These patients were divided into the isolated AFL and AFL combined with sporadic AF (AFL + paroxysmal AF [PAF]) groups, depending on whether they had coexistent PAF before TEE examination. PAF episodes were identified from all available 12-lead ECG recordings or 24-hour Holter monitoring records. Patients with no episode of PAF before TEE examination were classified into the isolated AFL group, and those with ≤2 episodes of PAF before TEE examination were classified into the AFL + PAF group. For comparison, patients who had paroxysmal AFL and sinus rhythm (SR) during TEE examination were included in the SR group as controls.

Data on demographics, personal health habits, medications, and medical history were obtained by reviewing the electronic medical records. This study protocol was reviewed and approved by the Research Ethics Committee of China Medical University Hospital (CMUH107-REC2–046). All study procedures were performed in accordance with relevant guidelines and regulations. The Research Ethics Committee waived the requirement for obtaining a signed consent form from all subjects because of the retrospective database research design of the present study.

The standard two-dimensional (2D) and Doppler transthoracic echocardiographic data (GE Vivid 7, General Electric, Milwaukee, WI, USA) and TEE (6VT-D ultrasound transducer, GE) data were retrospectively analyzed by two researchers who were blinded to the arrhythmia status and clinical characteristics. The diameter and volume of the LA were measured using both M-mode and 2D echocardiography. Echocardiographic examination of the left ventricular ejection fraction (LVEF), pulse waves, and tissue Doppler images was performed in accordance with standard protocols described previously^8,9^.

TEE images of multiple standard tomographic planes were analyzed to determine the peak LAA flow velocity (LAA-FV), LAA-EF, and the presence of an LAA thrombus or atrial SEC. An LAA thrombus was defined as a circumscribed and uniformly consistent echo-reflective mass that was different in texture from that of the wall of the LAA. SEC was defined as an intracavitary swirling smoke-like appearance in the LA or right atrium. The LAA-FV was determined by averaging the values from three consecutive cardiac cycles. The maximal and minimal LAA areas were measured by tracing a line starting from the top of the limbus of the left upper pulmonary vein along the endocardial border of the entire appendage^10^. The LAA-EF (%) was calculated using the following equation: (maximal LAA area − minimal LAA area) × 100/maximal LAA area.

Categorical data were presented as numbers (percentages) and continuous data as means ± SDs for normally distributed data or medians with IQRs for non-normally distributed data. Differences in demographic data, social histories, clinical characteristics, medications, and comorbidities among the three groups were examined using the ANOVA or Kruskal-Wallis test for continuous variables. The chi-square test was used to compare categorical variables. Regression analysis was used to analyze correlation between the CHA_2_DS_2_-VASc score and LAA-EF or LAA-FV in patients with isolated AFL and those with AFL + PAF.

All measurements were repeated by the same observer to determine the intraobserver variability, and the repeatability was analyzed using intraclass correlation coefficients (ICCs). The interobserver agreement between the two investigators (M-Y Wu and Y-N Lin) was measured using the ICC. A correlation coefficient of 0.21 to 0.40 indicates fair agreement; 0.41 to 0.60, moderate agreement; 0.61 to 0.80, substantial agreement; and ≥0.81, almost perfect agreement. Statistical analyses were performed using IBM SPSS Statistics version 22 (IBM, Armonk, NY, USA), and a P value of <0.05 was considered statistically significant.

## Results

Among 231 patients who received RFCA for IVC-TA-dependent AFL, 154 patients underwent TEE examination 1 day (IQR: 0−3 days) prior to RFCA (Fig. 1). Of these 154 patients, 14 patients with incomplete data on TEE were excluded. After excluding patients who met the exclusion criteria: AF rhythm during TEE examination (n = 37), presence of mitral stenosis or prosthetic valves (n = 11), ECG documentation of ≥3 episodes of AF before TEE examination (n = 7), and active cancer treatment (n = 1), a total of 84 patients constituted the study population for analysis. Of these 84 patients, 57 had ongoing AFL and were further divided into the isolated AFL group (n = 38) and AFL + PAF group (n = 19); the 27 remaining patients who had spontaneous SR at the time of TEE were designated as controls for comparison.

Table 1 shows the demographics and baseline clinical characteristics among the study patients. The mean patient age was 68.5 ± 13.5 years with male sex predominance (68.4%) in the patients with isolated AFL, which was similar to the patients with AFL + PAF (mean patient age: 63.3 ± 13.7 years; male sex predominance: 84.2%) at the time of TEE examination. The patients with isolated AFL had a mean body mass index of 24.2 ± 3.6 kg/m^2^, which was also similar to the patients with AFL + PAF (26.9 ± 5.2 kg/m^2^). The prevalence of major comorbidities, such as diabetes, hypertension, hyperlipidemia, myocardial infarction, heart failure, and stroke, was comparable among the three groups. The proportion of patients who were either current smokers or ex-smokers was higher in the AFL + PAF group (68.4%) than in the isolated AFL group (28.9%, P = 0.004). The proportion of patients with a CHA_2_DS_2_-VASc score of <2 (26.3% vs. 31.6%) or ≥2 points (73.7% vs. 68.4%) was similar between the isolated AFL and AFL + PAF groups.

**Table 1:** Demographics and baseline characteristics of the study patients.

Characteristics	SR (n = 27)	Isolated AFL^a^ (n = 38)	AFL + PAF^b^ (n = 19)	P1 value	P2 value (a vs. b)
Age (years)	59.7 ± 10.0	68.5 ± 13.5	63.3 ± 13.7	***0.023***	0.436
Sex				0.308	0.202
Male	22 (81.5%)	26 (68.4%)	16 (84.2%)		
Female	5 (18.5%)	12 (31.6%)	3 (15.8%)		
Body mass index (kg/m^2^)	26.4 ± 3.1	24.2 ± 3.6	26.9 ± 5.2	***0.019***	0.142
Current and ex-smokers	12 (44.4%)	11 (28.9%)	13 (68.4%)	***0.017***	***0.004***
Comorbidities					
Atrial fibrillation	15 (55.6%)	0	19 (100%)	—	—
Diabetes	5 (18.5%)	12 (31.6%)	6 (31.6%)	0.456	1.000
Hypertension	11 (40.7%)	20 (52.6%)	13 (68.4%)	0.180	0.255
Hyperlipidemia	6 (22.2%)	6 (15.8%)	5 (26.3%)	0.617	0.342
Ischemic stroke	0	1 (2.6%)	3 (15.8%)	***0.033***	0.067
Coronary heart diseases	3 (11.1%)	5 (13.2%)	3 (15.8%)	0.898	0.787
Heart failure	5 (18.5%)	14 (36.8%)	7 (36.8%)	0.237	1.000
Thyroid diseases	0	3 (7.9%)	2 (10.5%)	0.262	0.741
CKD/ESRD	2 (7.4%)	7 (18.4%)	2 (10.5%)	0.402	0.441
Cancer	1 (3.7%)	5 (13.2%)	2 (10.5%)	0.435	0.775
Medications					
Amiodarone	8 (29.6%)	10 (26.3%)	5 (26.3%)	0.951	1.000
Propafenone	3 (11.5%)	2 (5.3%)	3 (15.8%)	0.413	0.185
β-blockers	13 (48.1%)	15 (39.5%)	8 (42.1%)	0.782	0.849
Non-DHP CCBs	2 (7.4%)	5 (13.2%)	3 (15.8%)	0.653	0.787
Antiplatelets^*^	9 (33.3%)	18 (47.4%)	9 (47.4%)	0.479	1.000
Anticoagulants^¶^	11 (40.7%)	13 (34.2%)	9 (47.4%)	0.620	0.336
CHA_2_DS_2_-VASc score^#^	1 (0−2)	2.5 (1−4)	3 (1−4)	***0.004***	>0.999
<2	16 (59.3%)	10 (26.3%)	6 (31.6%)	***0.021***	0.677
≥2	11 (40.7%)	28 (73.7%)	13 (68.4%)		

Data are presented as mean ± SD or n (%). Continuous data were analyzed using the ANOVA and categorical data using the chi-square test. ^a^Isolated AFL; ^b^AFL + PAF; ^*^Aspirin, clopidogrel; ^¶^Warfarin, dabigatran, apixaban, edoxaban, and rivaroxaban. SR, sinus rhythm; AFL, atrial flutter; PAF, paroxysmal atrial fibrillation; CKD, chronic kidney disease; ESRD, end-stage renal disease; non-DHP CCB, non-dihydropyridine calcium channel blocker. P1: comparison among the three groups.

The comparisons of the conventional 2D echocardiographic data of the study patients are presented in Table 2. The LA diameter in the isolated AFL group (40.9 mm, IQR: 34.9−48.0 mm) was similar to that in the AFL + PAF group (41.4 mm, IQR: 32.8−49.3 mm, P > 0.999) and SR group (42.2 mm, IQR: 39.2−45.8 mm, P = 0.870). The LA volume was comparable between the patients with isolated AFL and patients with AFL + PAF (67.8 cm^3^, IQR: 48.5−88.4 cm^3^ vs. 56.7 cm^3^, IQR: 36.5−74.7 cm^3^, P = 0.311) and SR (63.7 cm^3^, IQR: 48.8−82.4 cm^3^, P = 0.363). The median LA volume index in the isolated AFL group was 37.1 cm^3^/m^2^ (IQR: 28.4−50.7 cm^3^/m^2^), which was not significantly different from that in the AFL + PAF group (34.4 cm^3^/m^2^, IQR: 19.6−42.6 cm^3^/m^2^) and SR group (34.3 cm^3^/m^2^, IQR: 24.3−42.1 cm^3^/m^2^). There was no significant difference in the systolic function (LVEF) of the left ventricle between the isolated AFL and AFL + PAF groups.

**Table 2:** Transthoracic echocardiographic parameters in the study patients.

Characteristics	SR (n = 27)	Isolated AFL^a^ (n = 36)	AFL + PAF^b^ (n = 17)	P1 value	P2 value (a vs. b)
IVSd (mm)	7.8 (6.8−8.8)	7.9 (6.9−9.6)	8.0 (6.9−9.8)	0.718	>0.999
IVSs (mm)	11.0 (10.4−12.7)	10.3 (9.6−12.2)	10.9 (9.9−12.2)	0.590	>0.999
LVIDd (mm)	52.8 (48.2−55.0)	49.8 (46.2−55.3)	53.7 (50.0−57.1)	0.158	0.109
LVIDs (mm)	33.3 (31.0−36.3)	34.1 (29.2−38.7)	36.2 (31.9−41.8)	0.270	0.255
LVEF (%)	57.3 (51.8−62.8)	55.8 (50.4−58.5)	53.1 (42.3−60.5)	0.236	>0.999
LA diameter (mm)	42.2 (39.2−45.8)	40.9 (34.9−48.0)	41.4 (32.8−49.3)	0.870	>0.999
LA volume (cm^3^)	63.7 (48.8−82.4)	67.8 (48.5−88.4)	56.7 (36.5−74.7)	0.363	0.311
LAVI (cm^3^/m^2^)	34.3 (24.3−42.1)	37.1 (28.4−50.7)	34.4 (19.6−42.6)	0.260	0.233
E/A	1.3 (0.9−1.5)	1.7 (0.9−2.6)	1.7 (0.7−2.4)	0.381	>0.999
E/e′	9.7 (7.0−14.1)	11.8 (9.0−14.1)	12.2 (9.8−18.8)	0.201	>0.999

Data are presented as median (IQR). Continuous data were analyzed using the Kruskal-Wallis test. ^a^Isolated AFL; ^b^AFL + PAF; IVSd, intraventricular septal end diastole; IVSs, intraventricular septal end systole; LVIDd, left ventricular internal diameter end diastole; LVIDs, left ventricular internal diameter end systole; LVEF, left ventricle ejection fraction; LA, left atrium; LAVI, left atrial volume index; E/A, ratio of the early (E) to late (A) ventricular filling velocities; E/e′, ratio of the mitral inflow early-diastolic velocity (E) to the septal and lateral mitral annulus spectral tissue Doppler early-diastolic velocities (e′). P1: comparison among the three groups

**Figure 2:**
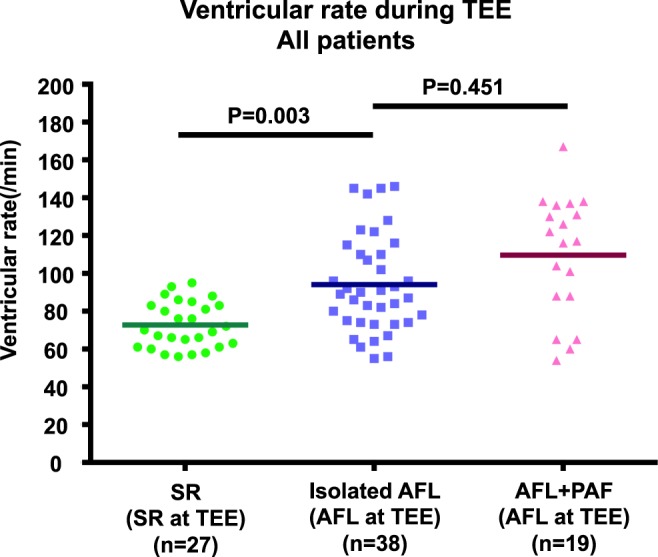
Ventricular rate during TEE in the study patients. There was no significant difference in the ventricular rate during TEE between the isolated AFL and AFL + PAF groups. TEE, transesophageal echocardiography; AFL, atrial flutter; PAF, paroxysmal atrial fibrillation.

**Figure 3:**
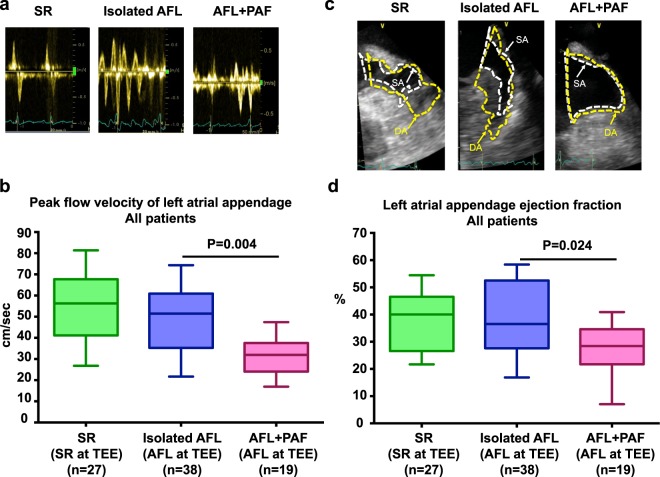
LAA-FV and LAA-EF in the study patients. (**a**) Diagram of LAA flow during SR or AFL rhythm in the representative patients. (**b**) Patients in the AFL + PAF group had a significantly lower LAA-FV than those in the isolated AFL group. (**c**) Visualization of the LAA in the representative patients using biplane TEE. The maximal (DA) and minimal (SA) areas of the LAA during one cardiac cycle are shown. The area of the LAA was measured by tracing a line starting from the top of the limbus of the left upper pulmonary vein along the entire endocardial border. (**d**) LAA-EF (%) = (DA–SA)/DA × 100. Patients in the AFL + PAF group had a poorer LAA-EF than those in the isolated AFL groups. LAA, left atrial appendage; LAA-FV, left atrial appendage flow velocity; LAA-EF, left atrial appendage ejection fraction; AFL, atrial flutter; SR, sinus rhythm; PAF, paroxysmal atrial fibrillation; TEE, transesophageal echocardiography.

There was also no significant difference in the ventricular rate during TEE between the isolated AFL and AFL + PAF groups (89.5/min, IQR: 74.0−111.3/min vs. 117.0/min, IQR: 88.0−136.0/min, P = 0.451; Fig. 2). The LAA-FV measured in the atrial systolic phase during AFL using TEE (Fig. 3) was significantly lower in patients with AFL + PAF than in those with isolated AFL (31.9 cm/s vs. 51.5 cm/s, P = 0.004; Table 3). Consistent with the LAA-FV, the LAA-EF measured during AFL using TEE (Fig. 3) was significantly lower in patients with AFL + PAF than in those with isolated AFL (28.4% vs. 36.5%, P = 0.024). Interestingly, the LAA-FV measured during AFL in the isolated AFL group was comparable to that measured during normal SR in the SR group (51.5 cm/s vs. 56.3 cm/s, P = 0.865). Similarly, the LAA-EF was equivalent between the isolated AFL and SR groups.

**Table 3:** LAA function and intracardiac thrombi in the study patients.

Characteristics	SR (n = 27)	Isolated AFL^a^ (n = 38)	AFL + PAF^b^ (n = 19)	P1 value	P2 value (a vs. b)
Ventricular rate (/min)	70.0 (61.0−83.0)	89.5 (74.0−111.3)	117.0 (88.0−136.0)	***<0.001***	0.451
LAA-FV (cm/s)	56.3 (41.2−67.7)	51.5 (35.3−60.9)	31.9 (24.1−37.5)	***<0.001***	***0.004***
LAA maximal area	5.3 (4.1−6.3)	3.7 (2.8−6.2)	4.5 (3.7−6.1)	0.052	>0.999
LAA minimal area	3.0 (2.5−4.4)	2.4 (1.5−4.2)	3.3 (2.1−4.7)	0.165	0.315
Difference between the LAA maximal and minimal areas	1.7 (1.5−2.5)	1.4 (1.0−3.1)	1.2 (1.0−1.4)	**<*0.001***	0.082
LAA-EF (%)	40.0 (26.6−46.5)	36.5 (27.6−52.5)	28.4 (21.7−34.6)	***0.016***	***0.024***
LAA thrombus	0	0	1 (5.3%)	0.177	0.154
LA SEC	5 (18.5%)	7 (18.4%)	4 (21.1%)	0.968	0.812
RA SEC	1 (3.7%)	1 (2.6%)	3 (15.8%)	0.118	0.067

Data are presented as mean ± SD, median (IQR), or n (%). Continuous data were analyzed using the Kruskal-Wallis test and categorical data using the chi-square test. ^a^Isolated AFL; ^b^AFL + PAF; LAA, left atrial appendage; LAA-FV, left atrial appendage flow velocity; LAA-EF, left atrial appendage ejection fraction; RA, right atrium; SEC, spontaneous echo contrast. P1: comparison among the three groups.

**Figure 4:**
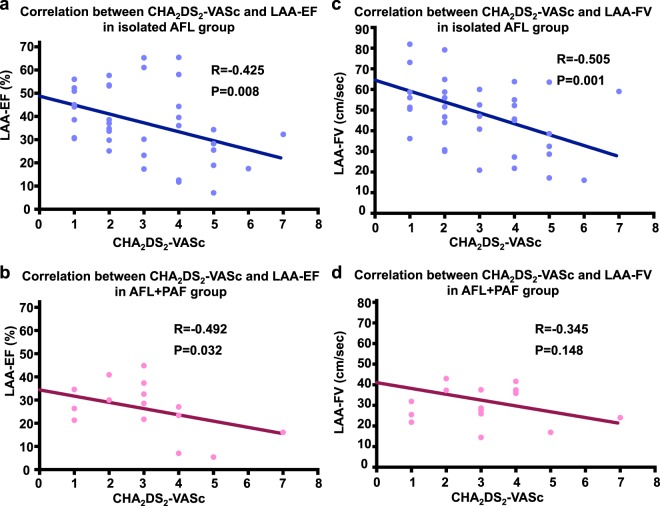
Correlation of the LAA-EF and LAA-FV with the CHA_2_DS_2_-VASc score. (**a**,**b**) Significant inverse correlations between the CHA_2_DS_2_-VASc score and LAA-EF were identified between patients with AFL and those with AFL + PAF. (**c**,**d**) An inverse relationship between the CHA_2_DS_2_-VASc score and LAA-FV was seen only in patients with isolated AFL not in those with AFL + PAF. LAA-FV, left atrial appendage flow velocity; LAA-EF, left atrial appendage ejection fraction; AFL, atrial flutter; PAF, paroxysmal atrial fibrillation.

We further compared the LAA function between the patients with isolated AFL and patients with AFL + PAF stratified by the CHA_2_DS_2_-VASc score (Fig. 4). Significant inverse correlations between the CHA_2_DS_2_-VASc score and LAA-EF were identified between the patients with AFL (R = −0.425, P = 0.008) and AFL + PAF (R = −0.492, P = 0.032) in the linear regression analysis. However, an inverse relationship between the CHA_2_DS_2_-VASc score and LAA-FV was seen only in the patients with isolated AFL (R = −0.505, P = 0.001), not in those with AFL + PAF (R = −0.345, P = 0.148). The results of negative correlations between CHA_2_DS_2_-VASc score and LAA-EF or LAA-FV remained valid after adjusting for the smoking status and ventricular rate during TEE by multivariate regression analysis (Supplementary Tables S1 and S2). The overall prevalence of LA thrombi was low in all study patients. There was only one patient found to have LA thrombus during TEE in the AFL + AF group. The prevalence of LA SEC was similar in the isolated AFL group and AFL + PAF group (18.4% vs. 21.1%, P = 0.812). The intraobserver ICC for the peak LAA flow velocities and LAA-EF was 0.883 (95% CI: 0.882−0.923) and 0.805 (95% CI: 0.708−0.873), respectively. The interobserver ICC and bias was 0.856, indicating an almost perfect agreement for both measurements between the two observers.

## Discussion

To the best of our knowledge, our study is the first to demonstrate that patients with AFL combined with rare, sporadic AF have a significantly poorer LAA function, as manifested by a depressed LAA-EF and lower LAA-FV, than those with isolated AFL, based on TEE imaging findings. Conversely, the LAA function measured during ongoing AFL in patients with isolated AFL was equivalent to that measured during SR in controls. Significant inverse correlations between the CHA_2_DS_2_-VASc score and LAA-EF were identified among patients with AFL and those with AFL combined with sporadic AF. These findings may help stratify the thromboembolic risk in patients with AFL.

The LAA is highly associated with intracardiac thrombus formation, especially in patients with nonvalvular AF^5^. An estimated 91% of intracardiac thrombi in nonvalvular AF occur in the LAA. The LAA flow velocity in patients with AF is significantly lower than that in those with SR^11^. A reduced LAA empty flow and an enlarged LA are highly correlated with thromboembolism in patients with nonvalvular AF^12^. Handke *et al*.^13^ reported that LAA flow velocities of <37 cm/s can predict the occurrence of stroke and that velocities of ≤55 cm/s are associated with a high risk of LAA thrombosis. Wai *et al*.^14^ reported that a reduced LAA blood flow velocity below 31 cm/s is an independent risk factor for the presence of SEC or thrombus formation in patients with AF. In addition to the LAA flow velocities, the LAA function can also be investigated on the basis of the LAA-EF^10,15^. Shimizu *et al*.^10^ reported that patients with AF and acute stroke have a significantly lower LAA-EF than patients with acute stroke and SR. Accordingly, exclusion of the diseased LAA through epicardial ligation may help improve the mechanical function of the LA, reverse LA remodeling, and contribute to lower thromboembolic risks in patients with AF^16^.

Apart from the established relationship between the LAA function and cardiogenic thromboembolism, Gupta *et al*.^17^ showed that abnormalities in the LA size and function, including LA emptying fraction and expansion index, are associated with a higher electrical burden of AF and greater risk of stroke. In the current study, as the LA diameter, LA volume, and LA volume index were equivalent among the three groups (SR, AFL, and AFL + PAF), we believe that assessment of the LAA function would be relevant to stratify the thromboembolic risks in this patient subset with comparable LA disease and low AF burden. Furthermore, previous studies have shown that LAA anatomies, including non-chicken wing shape of the LAA and higher LAA takeoff detected on computed tomography or magnetic resonance imaging, are associated with higher thromboembolic risks in patients with AF^18,19^. It would be interesting to know whether our patients with predominant AFL but carrying high-risk features for thromboembolism (low LAA-FV and LAA-EF and high CHA_2_DS_2_-VASc score) have similar LAA anatomies predisposing them to thromboembolism in future studies.

Compared with the well-established correlation between depressed LAA function and cardiogenic thromboembolism in AF, data on AFL appear heterogeneous and sometimes contradictory in previous studies^2,3,20^. Sakurai *et al*.^2^ divided the LAA flow velocity into <30 cm/s and ≥30 cm/s in 28 patients with AFL and found that a lower LAA flow velocity was associated with higher D-dimer and β-thromboglobulin levels, linking impaired LAA flow velocities with a prothrombotic state in patients with AFL. In general, the LAA velocity is higher in patients with AFL than in those with AF, as reported by Narumiya *et al*. (52.6 ± 18.2 cm/s vs. 31.6 ± 13.1 cm/s) and Huang *et al*. (63.3 cm/s vs. 44.4 cm/s)^3,7^; this indicates that patients with AFL, in general, may not be in the same prothrombotic state as those with AF.

In the current study, we first incorporated both the LAA-EF and LAA-FV to compare the LAA function between patients with isolated AFL and AFL + PAF. We further assessed the LAA function stratified by the CHA_2_DS_2_-VASc score between patients with isolated AFL and AFL + PAF. Significant inverse correlations between the CHA_2_DS_2_-VASc score and LAA-EF were identified between the patients with AFL and AFL + PAF. However, an inverse relationship between the CHA_2_DS_2_-VASc score and LAA-FV was seen only in the patients with isolated AFL, not in those with AFL + PAF. It has been shown that the LAA-FV is negatively correlated with the ventricular rate^19^. The lack of a significant inverse correlation between the CHA_2_DS_2_-VASc score and LAA-FV in the current study may be attributed, at least in part, to the high variations of the ventricular rate seen in the patients with AFL + AF. Nevertheless, our findings may provide additional information regarding the use of anticoagulation for the prevention of cardiogenic thromboembolism in patients with AFL or AFL combined with rare AF^21^. Indeed, further studies are needed to determine whether the LAA-EF and LAA-FV can be used to predict the stroke risk in patients with AFL.

We found that both the LAA-EF (36.5% vs. 28.4%) and LAA-FV (51.9 cm/s vs. 37.5 cm/s) were better in the patients with isolated AFL than in the patients with AFL and sporadic AF. We can argue reasonably that those with “rare” PAF had more left heart pathology than those with AFL, which was manifested in the way the LAA behaved during AFL in the current study. More detailed LA functional assessment, e.g., with strain measurements^22^, and LA fibrosis evaluation using cardiac magnetic resonance imaging may be needed in future studies. Nevertheless, the study findings support the concept that when PAF is present, though uncommonly, the thrombogenic risk in patients with isthmus-dependent AFL is higher than that in patients with isolated AFL alone based on the LAA functional characteristics.

Patients with AFL have been considered to have a similar thromboembolic risk as patients with AF based on limited data. However, patients with AFL may not be in the same prothrombotic state when compared with those with AF as previously thought^14^. The reported prevalence of LAA thrombi in patients with AFL varies from 0% to 11% among different populations studied^2,3,23,24^. We also found a low frequency of LAA thrombi in the patients with AFL + PAF (5.3%), which may be attributed to the low incidence of structural heart disease in our study patients. The low prevalence of LAA thrombus in the patients with AFL combined with sporadic AF may be attributed to the selection of patients with a truly rare AF event (median AF episode: 1) but not enrollment of patients with chronic or predominant AF as in previous studies. Besides, we did not exclude patients undergoing anticoagulation therapy (34.2% in the isolated AFL group and 47.4% in the AFL + PAF group), which may also reduce the incidence of intracardiac thrombi in the patients with AFL + PAF.

There are several limitations in our study. First, it investigated a selected patient population. We included only patients with ongoing AFL during TEE and those with typical IVC-TA isthmus-dependent AFL confirmed by an electrophysiological study. Thus, the results may not be generalizable to patients with atypical or other forms of AFL. Second, this study was a retrospective, single-center observational study with a small sample size, which may also lead to a selection bias. Further large-scale prospective studies are obviously required to confirm our findings. Third, we were unable to determine the exact onset and total duration of AFL because the time point of the first ECG record of AFL in our hospital might not be the first episode of arrhythmia. The total duration of AFL (short or long) may also affect the LAA function. Fourth, we did not measure the LA volume indices, including LA emptying fraction, LA expansion index, passive emptying fraction, and active emptying fraction, which could complement the current findings on LAA dysfunction. Fifth, although the anticoagulation status was comparable among the groups, the exact duration of anticoagulation therapy prior to TEE is not known, which might impact the echocardiographic prevalence of LA SEC or LAA thrombus in each group. In addition, we might have underestimated the short-lasting or asymptomatic AF episodes in the patients with sporadic AF. Similarly, some patients in the isolated AFL group may actually have silent or asymptomatic AF episodes not detected. Nevertheless, we have attempted to identify the AF event by reviewing all available ECG and Holter records to detect all AF events in both groups; we believe that this did not affect our findings on progressive deterioration of the LAA function between the patients with isolated AFL and AFL combined with only sporadic AF.

## Conclusions

The present study demonstrated progressive LAA dysfunction indicated by a significant reduction in the LAA-EF and LAA-FV in patients with AFL combined with sporadic AF compared with those with isolated AFL. The LAA-EF was inversely correlated with the CHA_2_DS_2_-VASc score, which may help stratify the thromboembolic risk in patients with AFL.

## Supplementary information

Supplementary Tables

## Data Availability

The data analyzed during the current study are available from the corresponding author on reasonable request.
