# Supplementary material for: Inverse Correlation Between Left Atrial Appendage Function and CHA2DS2-VASc Score in Patients with Atrial Flutter
Source: Sci Rep. 2019 Nov 28;9:17864. doi: 10.1038/s41598-019-54505-3 (PMC6883047; doi:10.1038/s41598-019-54505-3)
Supplement: Supplementary file 1 — Supplementary Tables [file 41598_2019_54505_MOESM1_ESM.pdf]

Inverse Correlation Between Left Atrial Appendage Function and CHA_2DS_2-VASc Score in Patients with Atrial Flutter

Mei-Yao Wu, MD, PhD, Yen-Nien Lin, MD, Hung-Pin Wu, MD, Ying-Ying Huang, MS, Jan-Yow Chen, MD, PhD, Kuo-Hung Lin, MD, Kuan-Cheng Chang MD, PhD

Supplementary Tables

Table S1. The correlation between LAA-EF and CHA_2DS_2-VASc

Groups	Covariates	Model 1		Model 2		Model 3	
		R	P	R	P	R	P
Isolated	LAA-EF	-0.425	0.008	-0.429	0.008	-0.460	0.006
AFL	Smoking	--	--	-0.116	0.448	-0.030	0.872
	Ventricular rate	--	--	--	--	0.152	0.432
AFL+PAF	LAA-EF	-0.492	0.032	-0.528	0.017	-0.619	0.008
	Smoking	--	--	-0.376	0.075	-0.363	0.080
	Ventricular rate	--	--	--	--	-0.273	0.201

Covariates for model 1: LAA-EF

Covariates for model 2: LAA-EF and smoking

Covariates for model 3: LAA-EF, smoking, and ventricular rate during TEE

Table S2. The correlation between LAA-FV and CHA_2DS_2-VASc

Groups	Covariates	Model 1		Model 2		Model 3	
		R	P	R	P	R	P
Isolated AFL	LAA-FV	-0.505	0.001	-0.520	0.002	-0.532	0.002
	Smoking	--	--	0.051	0.740	0.112	0.557
	Ventricular rate	--	--	--	--	0.101	0.581
AFL+PAF	LAA-FV	-0.345	0.148	-0.367	0.112	-0.396	0.102
	Smoking	--	--	-0.349	0.130	-0.339	0.149
	Ventricular rate	--	--	--	--	-0.145	0.533

Covariates for model 1: LAA-FV

Covariates for model 2: LAA-FV and smoking

Covariates for model 3: LAA-FV, smoking, and ventricular rate during TEE
